# Anomalous vascular perforator of the internal thoracic artery supplying a pedicled transverse rectus abdominis myocutaneous flap—a case report

**DOI:** 10.1093/jscr/rjac135

**Published:** 2022-05-23

**Authors:** Carlos Neblett, Leighton Logan, Kenneth Appiah, Kadeem Knight

**Affiliations:** Division of Plastic and Reconstructive Surgery, Kingston Public Hospital, Kingston, Jamaica; Division of Plastic and Reconstructive Surgery, Kingston Public Hospital, Kingston, Jamaica; Division of Plastic and Reconstructive Surgery, Kingston Public Hospital, Kingston, Jamaica; Division of General Surgery, Kingston Public Hospital, Kingston, Jamaica

## Abstract

The pedicled transverse rectus abdominis myocutaneous (TRAM) flap is an infrequently performed procedure relative to the more current microsurgical free tissue transfers in most centres around the world. However, in resource-limited centres where procedures requiring microsurgical intervention are rarely employed, the pedicled TRAM whose vascular axis is that of the superior epigastric artery, is an invaluable tool in the plastic surgeon’s armamentarium both for breast reconstruction and chest wall soft tissue resurfacing. This a report of a case of variable anatomical vascular perforating branch of the internal thoracic artery, which was encountered while using a TRAM to resurface a chest wall defect after mastectomy was performed for locally advanced breast cancer.

## INTRODUCTION

Breast cancer is the most prevalent cancer in women with a lifetime incidence of one in eight-nine [1]. Surgical treatment for breast cancer is usually in the form of mastectomies as they account for about 45% of these procedures [2]. Surgical extirpation employed for locally advanced breast cancer typically culminate with large chest wall defects requiring appropriate and adequate coverage using either soft tissue flaps or skin grafts. Local fasciocutaneous and regional myocutaneous flaps (latissimus dorsi, rectus abdominis) are performed due to their aesthetically pleasing durability, and if adjuvant radiation therapy is needed [3]. The authors present a case of locally advanced breast cancer post-preoperative chemoradiation therapy managed with a pedicled transverse rectus abdominis myocutaneous flap, which was found to have an anomalous perforating branch from the internal thoracic artery, which arose deep to the posterior rectus sheath. We are unaware of this anatomical variation being documented to the best of our knowledge.

## CASE REPORT

A 45-year-old afro-Caribbean woman presented with a two-year history of an initially slow growing left breast lump, which then rapidly increase 6 months prior to presentation with associated nipple and skin changes. No constitutional symptoms were reported and her medical history was remarkable for an exploratory laparotomy due to an ectopic pregnancy via a Pfannensteil incision.

Clinical examination revealed a woman with a body mass index of 25.9 kg/m^2^ and 8.0 × 8.0 cm fungating mass in the upper outer quadrant with peau d’orange and nipple areolar retraction along with clinically palpable ipsilateral axillary lymphadenopathy.

A core needle biopsy was performed and the histopathology confirmed invasive ductal carcinoma with the immunohistochemistry revealing oestrogen, progesterone, human epidermal growth factor receptors (triple) negative disease. Staging computed tomographic scans of the chest, abdomen and pelvis showed no evidence of metastatic disease. The diagnosis according to the American Joint Committee on Cancer 8th Edition was Stage IIIB Invasive Ductal Adenocarcinoma.

Preoperative chemotherapy was commenced using adriamycin, cyclophosphamide and taxol with complete clinical response of the breast and axillary disease and the patient defaulted from surgical outpatient department follow-up.

She represented seven months later with a recurrent left breast mass but no axillary masses. Repeat biopsy confirmed the previous histological findings with staging scans showing no evidence of distant spread. Chemo-radiation therapy was initially administered with no clinical response. A multidisciplinary team meeting consensus was for total mastectomy with level II ipsilateral axillary dissection and soft tissue resurfacing with an ipsilateral pedicled transverse rectus abdominis myocutaneous (TRAM) flap.

**Figure 1:**
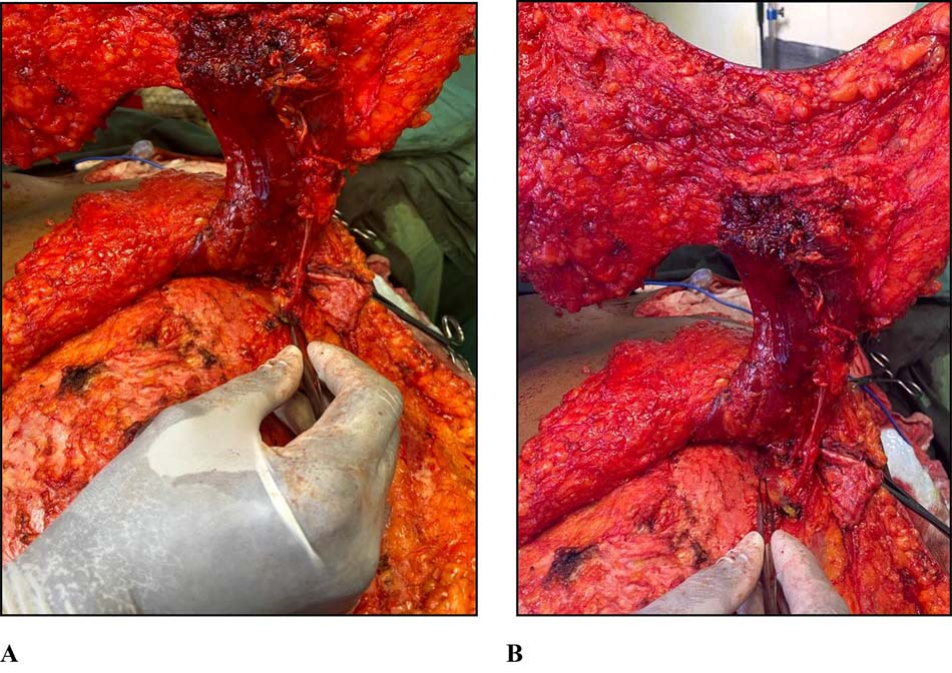
(**A**) Photograph of forceps pointing to the perforating vascular pedicle arising from the left transversus abdominis muscle and supplying the rectus abdominis muscle. (**B**) Photograph of forceps pointing to the left transversus abdominis muscle.

There was a 20 × 20 cm anterior thoracic wall defect after extirpative mastectomy and resurfaced with the TRAM flap. The flap was harvested from caudal to cranial and the superior epigastric vascular pedicle was found to be running on the posterior surface of the rectus abdominis muscle. However during further dissection towards the costal margin, we observed an atypically located perforating branch of the internal thoracic artery arising deep to the posterior rectus sheath in the substance of the transversus abdominis muscle. The pedicle was mobilized from the transversus abdominis muscle (Fig. 1), allowing the TRAM flap to be delivered from the abdomen to the thorax via the subcutaneous tunnel in a tension free manner facilitating defect resurfacing. Flap inset was achieved using a two layered closure while the abdominal wall was reconstructed using a polypropylene mesh followed by closure and in-setting of the umbilicus. Two closed active drains were left in situ in the anterior thoracic and abdominal walls. The patient was discharged on postoperative day 6 with unremarkable recovery.

## DISCUSSION

Surgical ablation of locally advanced breast cancer requires significant excision to minimize oncologic recurrence thereby resulting in large anterior chest wall defects. Reconstructing these defects typically means employing robust flaps such as the pedicled latissimus dorsi myocutaneous, the pedicled or the free transverse rectus abdominis myocutaneous and the free deep inferior epigastric artery perforator flaps due to them being more aesthetically acceptable and being able to withstand the rigours of radiotherapy [4]. The ipsilateral pedicled TRAM flap was chosen to reconstruct the defect because the thoracodorsal vascular pedicle was previously exposed to radiation and raising a flap on this vascular axis was deemed to be precarious in our opinion. Additionally, in our resource-limited centre microsurgical procedures are significant time consuming undertakings and with the minimal operating time afforded such procedures would not be the best use of our resources.

Hartrampf described the pedicled transverse rectus myocutaneous flap in 1981 as a means for breast mound reconstruction specifically, though its use has also been adapted as one of the options for chest wall reconstruction [5]. This flap utilizes the redundant excess lower abdominal tissue typically excised during abdominolipectomy to recreate the chest wall soft tissue defect and is based on the superior epigastric artery (SEA), which carry some of the blood supply to the rectus abdominis muscle. It is pivoted on the rectus in order to transpose the lower abdominal soft tissue in an islanded fashion to the chest wall defect using a subcutaneous tunnel from the upper abdomen to the chest wall, which allows the passage of the flap into the defect site [6].

Based on anatomical studies, the SEA traverses inferiorly along the same line as the internal thoracic artery (ITA) coursing anterior to the diaphragm, transversus thoracis and transversus abdominis in Larrey’s space continuing on the posterior surface of the rectus abdominis anterior to the rectus sheath before entering the muscle and supplying it, then anastomosing with the inferior epigastric artery in the region of the umbilicus [7, 8]. In this report, an anomalous perforating branch, possibly arising from the ITA or the uncommon xiphoid branch variation of the ITA was noted deep to the posterior rectus sheath within the substance of the transversus abdominis muscle from which it was dissected facilitating flap viability and ensuring tension-free delivery from the donor to the recipient site [9, 10]. Cadaveric studies are necessary to elucidate the prevalence of this anomaly. We believe that this variation should be borne in mind to ensure successful flap harvest when employed for breast or chest wall reconstruction.
